# Nutrition security, constraints, and agro-diversification strategies of neglected and underutilized crops to fight global hidden hunger

**DOI:** 10.3389/fnut.2023.1144439

**Published:** 2023-06-22

**Authors:** Akib Ali, Bijoya Bhattacharjee

**Affiliations:** Division of Crop Science, ICAR Research Complex for NEH Region, Umiam, Meghalaya, India

**Keywords:** nutrition security, neglected and underutilized crops, bioactive components, anti-nutritional factors, climate resilience

## Abstract

**Introduction:**

Neglected and underutilized crop species (NUCS) or forbidden crops offer tremendous potential to combat malnutrition, poverty, and global hidden hunger. Since overdependence on a few dominant cereal crops, *viz.,* rice, maize, and wheat, is insufficient to meet the global food energy intake, the identification, genetic improvement, and implementation of various policies for wenumerates comprehensive comparative analyses of the nutrient profile of staple crops vs. potent underutilized crops with reference to cultivation constraints and climate resilience with different agro-diversification strategies.

**Methodology:**

The research databases Scopus, JSTOR, Web of Science, EBSCO, Google Scholar, ScienceDirect, PubMed, and Academic Search were searched using relevant research queries.

**Result:**

Out of 2,345 hits, 99 articles pertinent to the subject domain showed that underutilized crops are nutritionally superior, contain health-promoting bioactive components, and are more climate resilient than cereal crops. However, several constraints hinder the efficient utilization of these crops.

**Discussion:**

Despite underutilized crops’ many health benefits, improved cultivation techniques for the large-scale production of these crops are still in their infancy. Most of the time, however, the scientific knowledge gleaned from various study domains stays within the scientific community. The most crucial need of the hour, therefore, is an efficient network structure connecting governments, farmers, researchers, and people in business. Moreover, care must be taken to ensure that the policies of governments and INGOs/NGOs are properly implemented within a NUCS framework.

## Introduction

**Figure 1:**
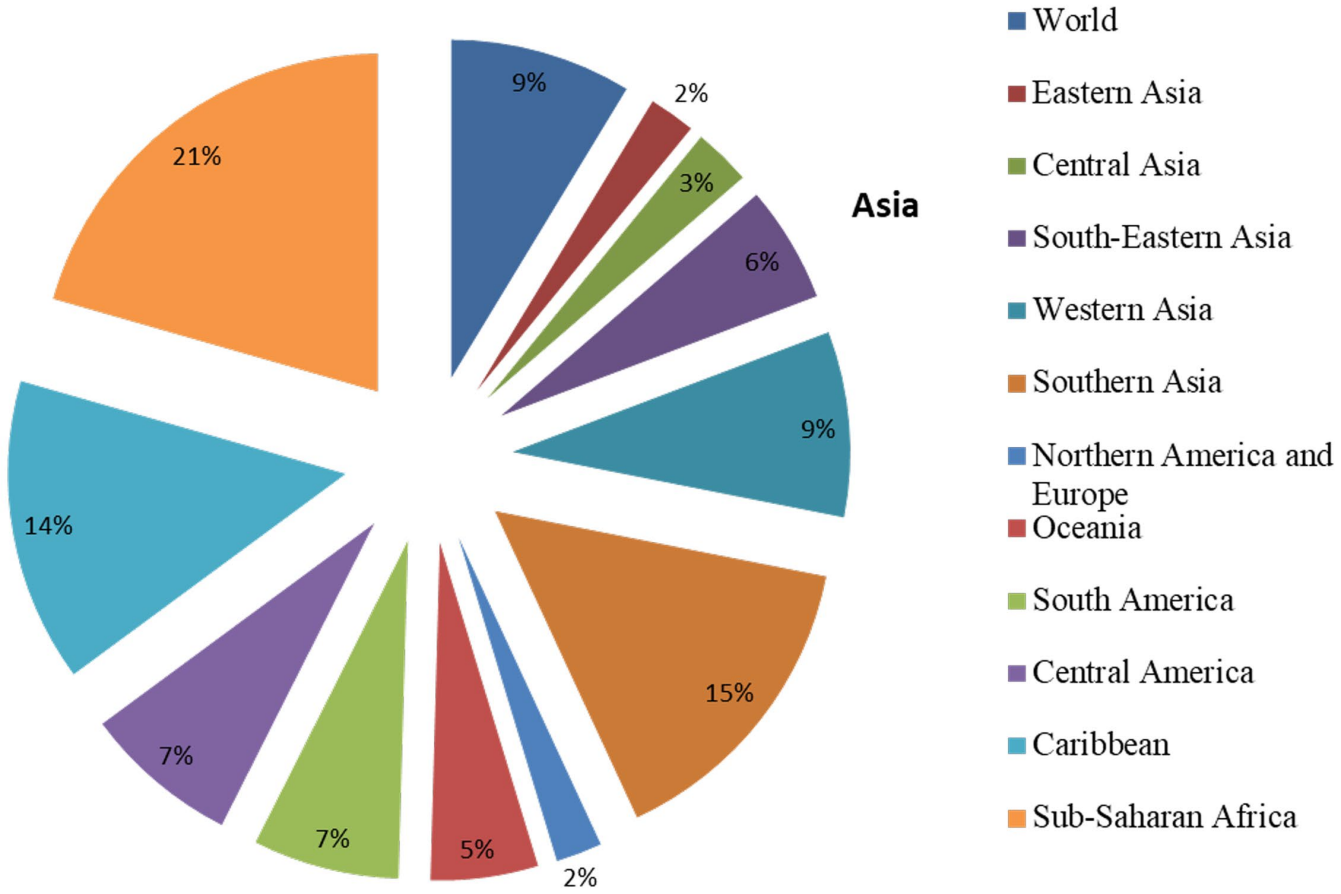
Region-wise share of population under malnutrition (Adapted from Statista 2022).

An endeavor to improve global food security is being hindered by several intricate and interlinked challenges. Especially after the COVID-19 crisis, development across many domains is either being halted or reversed, worsening an already dire scenario that includes hunger and food insecurity. Even though the world has already fallen behind schedule in achieving the Sustainable Development Goals (SDGs) before 2020, the pandemic has accelerated this trend and had a disastrous impact on individual lives and livelihoods as well as global efforts to achieve the SDGs. The Food and Agriculture Organization (FAO) of the United Nations recently estimated the global hunger figure to be approximately 702 to 828 million people (1). Additionally, drastic food insecurity has increased significantly from 10% (2020) to 11.7% (2021) of total world population (1). According to the Global Hunger Index 2022, 44 countries are experiencing an alarming level of hunger issues, and 20 more countries showed a higher GHI score than in 2014, with moderate, *se*rious, or alarming hunger levels, while 46 countries will fail to reduce hunger level by 2030. Among different countries, Chad, Madagascar, the Central African Republic, Yemen, Somalia, Syria, South Sudan, Congo, and Burundi showed GHI scores ranging from 37.2 to 45.1. A recent report by Statista 2022 revealed that nearly 23.2% of the population of Sub-Saharan African is experiencing malnutrition, while North America, Europe, and East Asia have the lowest (2.5%) share of population experiencing malnutrition (Figure 1). These differences could be attributed to inefficient food supply, low income, and poor health facilities in poor/developing countries as compared to developed countries. It is evident that more investment in the agricultural sector than non-agricultural sectors is highly effective in reducing poverty and global hunger. While more than 700 million hectares of global cropping areas are used to cultivate the five major cereal crops of maize, wheat, rice, barley, and sorghum, which alone supply 50% of the world’s caloric intake (2), yield and grain quality have been plateauing in recent years, with a substantial reduction since 1960s (3). The dilemma of food insecurity cannot be met by focusing on just the productivity of the existing primary crops, which have been frequently chosen and developed under high-intensity agriculture. This could also render agriculture even more susceptible to future biotic and abiotic pressures. Over the past decade, research on potent alternate crops, *viz.*, neglected and underutilized crop species (NUCS), has gained considerable ground due to the focus on food quality, reduction of the risk of overreliance on a limited number of staple crops, preservation of cultural dietary diversity, and the potential of natural climate-resilient crops (4, 5). However, not every neglected and underutilized crop species (NUCS) is climate resilient and nutrient rich. The FAO (6) classifies NUCS as Future Smart Food (FSF) on the condition that they satisfy the following four criteria: they must be nutrient rich (improve nutrition), climate resilient (e.g., enhance climate change resiliency and environmentally sustainability by effectively reducing runoff and erosion), locally available or adaptable, and cost effective (e.g., produce income and lessen drudgery). In the present review article, we have provided a comprehensive comparative nutrient profile of staple crops vs. potent underutilized crops with reference to their climate resilience. Major bioactive components, bioavailability and anti-nutritional factors, and different constraints of NUCs were also briefly discussed. The detailed descriptions of the methodology and information sources are shown in Supplementary Note 1.

### Identification and comparative nutritive values of neglected and underutilized crop species

NUCS, also known as forbidden or orphan crop species, are indigenous to a particular tribe, usually semi-domesticated or wild. NUCS are classically identified based on the following features described by Papoola et al. (7) and Chandra t al. (8):

1. Crops that have known native uses in specific localized areas with scientific or ethno-botanical evidence of their nutritional value.
2. Adaptation to agro-ecological niches or marginal areas and representation by ecotypes/landraces.
3. These crops must be grown less frequently as compared to traditional staple crops.
4. Adaptation and cultivation are based on indigenous knowledge and practices.
5. Rare depiction in the *ex situ* collection.
6. Grain supply networks are either underdeveloped or non-existent.
7. They have received scant attention from scientists, extension agents, farmers, policymakers, technologists, and consumers.
8. These crops might be extremely nutritive and/or possess therapeutic potential with other multiple uses.

NUCS include species from all forms of plants, *viz.*, herbs, shrubs, trees, crops, or vines. However, due to the wide range of species—and with the definition depending on the location, scale of reference, and possibility for enhanced use—compiling a comprehensive list of neglected and underutilized crops is not an easy process (9). The available lists as reported by the National Research Council of Africa (10), Li et al. (11), Wani et al. (12), and Hossain et al. (13) for Africa, Asia, and the Americas are subjected to regional preferences. A compiled list of potent NUCS has been shown in Supplementary Table 1. Among the different crops, the recent emergence of crops such as amaranth, buckwheat, yam, Colocasia, lemon, pumpkin, cassava, faba beans, millets, legumes, pulses, and traditional vegetables has diversified the food consumption profile of marginalized and neglected crop species. Although these crops are suitable for marginal areas, they still hold a significant value in the local food basket due to their sufficient nutrient content (14). A detailed nutrient profile of major staple crops and some specific underutilized crops has been shown in Table 1. Millets are highly nutritious and easily digestible due to their low glycemic index (17) compared to the staple cereals, and they are reported to be the sixth highest yielding crop in the world (18). While finger millets contain >10 times higher calcium content than polished rice (19), Quinoa (*Chenopodium quinoa*), a pseudocereal, is a rich source of protein and fiber (20), contains 14% protein, essential amino acids, a rich source of vitamins, fatty acids, and is free of cholesterol and gluten (20). In comparison to cereals, pseudocereals have significantly high-quality protein, which makes them appropriate for the functional food market. Pseudocereals are abundant in amino acids, including arginine, tryptophan, lysine, and histidine, which have been shown to be crucial for newborn and child health. This has led to the projection of pseudocereals as a suitable food supplement for child nutrition. The essential amino acid, *viz.*, lysine, content in buckwheat is much higher compared to rice, wheat, or maize. A comparative list of amino acid contents between cereals and NUCS is presented in Supplementary Table 2. Buckwheat is also a rich source of flavonoids such as rutin, quercetin, and alpha tocopherol (21). As per the FAOSTAT, 2018 report, France was reported to be the highest producer of buckwheat in the world (3,735 kg/ha). Grains of Amaranthus contain 64% starch, 10% fat, 4% protein, 2.5% ash, 16% fiber content, and essential amino acids such as lysine (22). The cultivation of underutilized pulses such as mung beans, pigeon peas, or lentils is confined to mostly Southeast Asian countries, and their production level is still considered insignificant compared to major cereals. While sprouted Adzuki beans (*Vigna angularis*) are a rich source of vitamin A, vitamin B, and folic acid with 19.9% protein content (23), jack bean is an excellent source of protein (23–34%) and carbohydrate (55%) (24). *Portulaca oleracea* contains 26.6 mg of ascorbic acid, 300–400 mg omega-3 fatty acids, 1.9 mg beta-carotene, 12.2 mg of α-tocopherol, and 14.8 mg of glutathione (25). Among many underutilized leafy vegetables, amaranth is widely grown in tropical regions such as Mexico, South America, Southern Asia, and Africa. Compared to cabbage, it is significantly more nutritious (26). Another nutrient- and mineral-rich underutilized plant is the drumstick, mostly cultivated in tropical Asia, Sub-Saharan Africa, Latin America, and the Caribbean. According to Chandrashekara and Kumar (27), root and tuber crops (RTCs) are the second highest source of carbohydrates after cereals. The fact that RTCs contribute significantly to global food security and are produced at >845 million tons on a global scale (6) demonstrates their significance. Yam is the fourth most produced RTC in the world, and it is primarily produced and consumed in Southeast Asia, West Africa, and the Caribbean (28). Sweet potato, cassava, yams, and aroids, rather than potatoes, account for 90% of production and consumption among the various underutilized roots and tuber crops. Currently, the largest producer of these crops is Asia, followed by America and Europe (28). Asia alone contributes more than 40% of global production. Cassava is a potent NUCS for more than 500 million people worldwide due to its distinctive nutritional components and high carbohydrate content (29). Recently, the concept of dietary fiber has attracted worldwide attention. Underutilized vegetables are considered reservoirs of various minerals. The nutritious pods of *Parkia roxburghii* and *Mucuna Pruriens* are considered among the most popular legumes in Northern India (30).

**Table 1:** Nutrient profile of selected staple cereal crops vs. neglected and underutilized crop species (NUCS) (11, 13, 15, 16).

Crop Categories		Scientific name	Nutrients composition (g/100 g)						
			Common name	Energy	Protein	Carbohydrate	Fiber	Fat	Ash
Staple Crops	Cereals	*Oryza sativa*	Milled rice	345	6.8	78.2	4.1	3.6	0.8
		*Oryza sativa*	Red rice	362	7.5	76.2	3.6	2.4	1.5
		*Triticum aestivum*	Wheat	344	11.8	71.2	12.2	2.73	0.9
		*Zea mays*	Maize	366	9.4	63.6	7.3	4.7	1.78
Neglected and Underutilized Crop Species (NUCS)	Cereals and Pseudocereals	*Eleucine coracana*	Finger millets	328	7.3	75	20	1.3	1.9
		*Setaria italic*	Foxtail millet	331	12.3	69.9	4.25	4.3	1.9
		*Pennisetum glaucum*	Pearl millets	361	11.6	61.78	11.49	5	1.9
		*Fagopyrum esculentum*	Buckwheat	355	14.2	72.9	17.8	7.4	2
		*Amaranthus caudatus*	Amaranth	346	14.5	63	12.5	2.5	1.8
		*Chenopodium quinoa*	Quinoa	354	14.1	57.16	7	4.7	1.8
		*Perilla frutescens*	Perilla	544	17	44.1	3.2	51.7	3.7
	Root and tubers	*Colocasia esculenta*	Cocoyam	112	1.5	85.36	4.1	2	2.27
		*Ipomoea batatas*	Sweet potato	86	1.6	20.12	3	4.7	1.37
		*Solanum tuberosum*	Potato	95	2.63	21.4	2.3	0.13	1.3
		*Manihot esculenta*	Cassava	160	1.36	38.1	1.8	0.28	2.3
	Vegetables and pulses	*Amaranthus dubias*	Amaranth	49	4	46	2.87	0.2	2.3
		*Brassica oleracea*	Brassica	21	9	6	1	1	2.4
		*Lablab purpureus*	Hyacinth beans	344	23.9	60.74	25.6	1.69	0.7
		*Vicia faba*	Broad beans	341	26.12	58.59	25.0	1.53	1.7
		*Parkia roxburghii*	Tree bean	426	18.8	39.74	9.56	15.5	4.1
		*Vigna umbellate*	Rice beans	348	20.9	60.7	4.0	0.9	2.4
		*Moringa oleifera*	Moringa leaf	92	6.7	13.4	1.7	1.7	0.9
		*Moringa oleifera*	Moringa pod	26	2.5	3.7	0.1	0.1	4.8
		*Vigna angularis*	Adzuki beans	412	20	6.0	13.0	0.5	1.4
		*Psophocarpus tetragonolobus*	Winged beans	183	40	45	7	20	2.1
		*Canvalia ensiformis*	Jack beans	241	30	54.28	9.9	7.1	1.9
		*Coccinia Grandis*	Ivy guard	21	15	12.62	3.4	4	0.8
		*Nelumbo nucifera*	Indian lotus	350	15	65	1.9	2	1.5
		*Sechium edule*	Chow	19	0.82	4.51	1.7	0.13	0.9
		*Citrullus lanatus*	Watermelon	296	3.5	8	3.8	0.4	3.8
		*Lagenaria siceraria*	Bottle guard	14	0.62	3.7	0.5	0.02	0.5
		*Solanum dulcamara*	Nightshade	55	3	74	2.42	0.6	2.24
		*Corchorus olitorius*	Jews mallow	392	20.9	12.2	45.61	5.2	0.16
		*Cicer arietinum*	Chickpea	1,201	17.1	60.9	3.9	5.11	1
		*Phaseolus vulgaris*	Kidney beans	1,245	22.9	60.6	4.8	1.77	0.6
		*Lens culinaris*	Lentils	1,349	25.1	59	0.7	0.75	0.8
		*Glycine max*	Soybeans	1,597	43.2	20.9	3.7	19.42	1.2
		*Vigna radiate*	Green gram	1,363	24	56.7	4.1	1.14	0.9
		*Pisum sativum*	Pea	1,269	72	15.9	4	1.89	1
	Fruits and Nuts	*Phoenix dactylifera*	Dates	301	5.1	62.2	8.4	9	0.7
		*Annona squamosal*	Annona	113.65	1.25	18.65	21.62	3.78	0.6
		*Passiflora edulis*	Passion fruit	97	2.2	23.38	10.40	0.7	0.5
		*Carica papaya*	Papaya	32	0.6	7.2	2.6	0.1	0.2
		*Artocarpus heterophyllus*	Jackfruit	95	1.72	23.25	1.5	0.64	2.1

### Major bioactive components of NUCS and their health benefits

**Figure 2:**
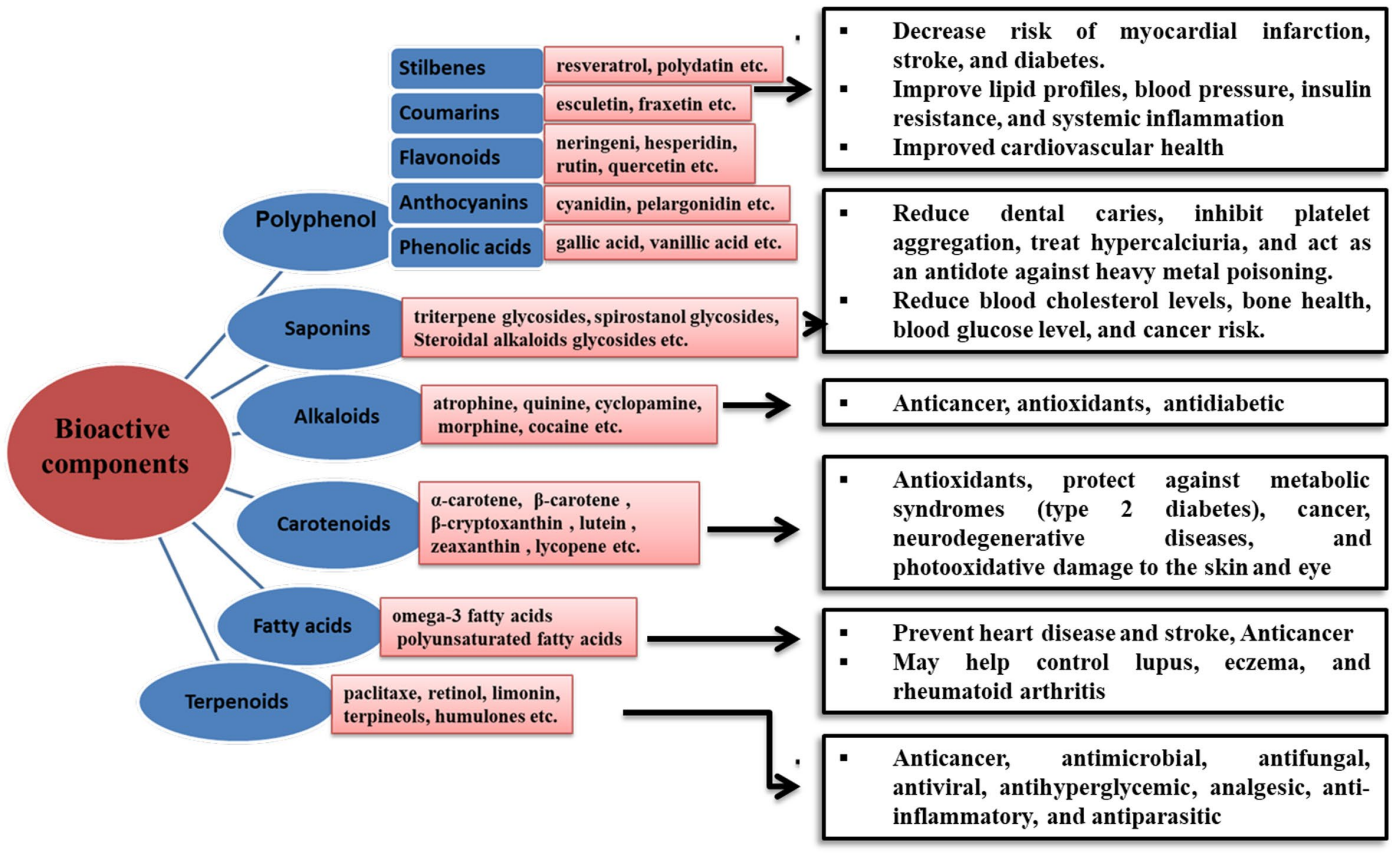
Schematic representation of bioactive components found in NUCS and their health benefits.

Bioactive components in plants can be classified into four categories, *viz.*, phenolic acids, flavonoids (anthocyanidins, flavones, isoflavones, flavonols, and flavanones), stilbenes, and lignans. These naturally occurring antioxidants, especially flavonoids, have a wide range of biological activities, including anti-aging, anti-inflammatory, antiviral, antimicrobial, and anti-cancer properties. Different bioactive components and their health benefits have been shown in Figure 2. Several studies have reported that neglected and underutilized crops are rich sources of bioactive components (for example, bioactive flavonoids present in different parts of pseudocereal buckwheat, *viz.*, the root, flower, fruit, seed, sprouted seed, seedling, seed coat, seed husk, and processed food, establishing it as a highly treasured crop) (31). Rutin comprises 90% of total flavonoid phenolics, followed by quercetin (32, 33). High phenolic content imparts higher antioxidant activity in buckwheat than quinoa and amaranth (34). It has been reported that nitrogen-containing pigments such as betalains exist more abundantly in pseudocereals than in cereals. On the other hand, flavonoids and carotenoids are the major bioactive components present in fruits and vegetables. Some significant bioactive components found in the different underutilized crops, *viz.*, pseudocereals, fruits, vegetables, roots, and tubers crops, are listed in Table 2.

**Table 2:** Bioactive components found in neglected and underutilized crop species (NUCS).

Scientific name	Common name	Bioactive components	References
*Eleucine coracana, Setaria italic,Pennisetum glaucum*	Finger millet, Foxtail millet,Pearl millet	Phenolic acids, anthocyanins, tannins, pinacosanols, catechin, epicatechin, quercetin, apigenin, hydroxybenzoic acid, protocatechuic acids, p-hydroxybenzoic acids, syringic acids, ferulic acid, and hydroxycinnamic acid	(35–37)
*Fagopyrum esculentum*	Buckwheat	Syringetin, dihydromyricetin, kaempferol, kaempferide, isorhamnetin, myricetin, quercetin, kaempferol, kumatakenin, fustin, laricitrin, morin, syringetin, isorhamnetin, afzelechin, hesperitin, naringenin, orientin, vitexin, homoorientin, and isovitexin	(38)
*Amaranthus caudatus*	Amaranth	Vanillic, 4-hydroxybenzoic, 4-syringic, caffeoylisocitric acids, rutin, isoquercitrin, α-tocopherol, β-tocotrienol, γ-tocotrienol, and δ-tocopherol	(39)
*Chenopodium quinoa*	Quinoa	Carotenoids (lutein, zeaxanthin, beta-carotene, and lutein) and phenolic acids	(39)
*Perilla frutescence*	Perilla	Rosmarinic acid, caffeic acid, and anthocyanins	(40)
*Colocasia esculenta*	Cocoyam	Alkaloids, tannins, flavonoids, saponins, polyphenols (flavonoids and phenolic acids), anthraquinones, and dioscorin and diosgenin	(41)
*Ipomoea batatas*	Sweet potato	Vitamins, amino acids and minerals, tocopherol, and beta-carotene	(42)
*Solanum tuberosum*	Potato	Polyphenols, anthocyanins, flavonoids, carotenoids, tocopherols, and vitamin C	(43)
*Manihot esculenta*	Cassava	Flavonoids, β-carotene, saponins, tannins, sitosterol, and stigmasterol	(44)
*Lablab purpureus*	Hyacinth beans	Phenols, steroids, essential oils, alkaloids, tannins, flavonoids, saponins, coumarins, terpenoids, glycosides, and anthocyanin	(45)
*Vicia faba*	Broad beans	Flavonoids, lignans, and terpenoids; protocatechuic acid, ferulic acid, vanillic acid, caffeic acid, sinapic acid, salvianolic acid, *cis*- and *trans*-*p*-coumaric acid, hydroxyeucomic acid, eucomic acid, caffeoylquinic acid, and dicaffeoylquinic acid	(46)
*Macrotyloma uniflonum*	Horse gram	Alkaloids, flavonoids, saponins, phenols, glycosides, tannins, terpenoids, quinones, mome inositol, hexadecanoic acid, methyl ester, octadecanoic acid, and gamma tocopherol (46)	(47)
*Mucuna pruriens*	Velvet beans	L-3,4-dihydroxyphenylalanine, lectin, isoflavanones, and some alkaloids, phenol, flavonoid, proanthocyanidin, and rutin	(48)
*Moringa oleifera*	Moringa leaf	Vitamins, carotenoids, polyphenols, phenolic acids, flavonoids, alkaloids, glucosinolates, isothiocyanates, tannins, and saponins	(49)
*Fabaceae*	Legumes	Polyphenols, alkaloids, saponins, carotenoids, terpenoids, omega-3 fatty acids, flavonoids, and anthocyanins	(50)
*Coccinia Grandis*	Ivy guard	Lupeol, β-sitosterol, β-amyrin, coccinioside-k, stigmast-7-en-3-one, flavonoid glycoside, phenol, benzofuranone, hexadecanoic acid methyl ester, β-sitosterol acetate, tocopherol, stigmasterol, ethisteron, and campesterol	(50)
*Nelumbo nucifera*	Indian lotus	Alkaloids, flavonoids, glycosides, triterpenoid, and vitamins	(51)
*Sechium edule*	Chow	C-glycosyl, O-glycosyl flavones, lutein, and β-carotene, tocopherol, myricetin, ferulic acid, chlorogenic acid, and (+)-catechin	(52)
*Solanum dulcamara*	Nightshade	Sugars, vitamin C, vitamin E, polyphenols, and flavonoids	(53)
*Lens culinaris*	Lentils	trypsin/protease inhibitors, lectins, defensins, polyphenols, flavonoids, triterpenoids, saponins, phytates, and phytosterols	(54)
*Glycine max*	Soybeans	Isoflavones, saponins, phytic acids, phytosterols, trypsin inhibitors, and peptides.	(54)
*Phoenix dactylifera*	Dates	Protocatechuic, gallic, caffeic, p-hydroxybenzoic, vanillic, ferulic, syringic, p-coumaric, o-coumaric acid, carotenoids, and flavonoids	(55)
*Annona squamosal*	Annona	Sodium benzoate, 4, 4-tert-butylcalix(4)arene, 4, 4-dimethylcholesterol, butyl octyl phthalate, stigmasterol acetate, and isoamyl acetate	(56)
*Passiflora edulis*	Passion fruit	C-glycosyl flavonoids vicenin, orientin, isoorientin, and vitexin	(57)
*Carica papaya*	Papaya	Alkaloids, flavonoids, polyphenols, and fatty acids	(58)
*Artocarpus heterophyllus*	Jackfruit	Phenolics, flavonoids that comprise prenylflavonoids, hydroxycinnamic acids, and glycosides, stilbenoids, triterpenoids, and steroids	(59, 60)

### Climate-resilient crops: staple crops vs. NUCS

Global climate change has threatened the productivity of major staple crops such as rice, wheat, and maize. Cereal crops are sensitive to various biotic and abiotic constraints, *viz.*, salinity, drought, or heavy metal stresses. Under enduring climate change and variability conditions, underutilized crops are regarded as mitigation strategies for food insecurity (61). A considerable decrease in wheat output has been anticipated to occur in temperate and tropical locations with every 2°C increase, as depicted by a meta-analysis of 1,700 published models (62). Similar climate modeling studies forecast a 6% drop in wheat yield, which translates to a potential 42 Mt./°C drop (63). Based on simulations using the regional calibrated crop model, Lu et al. also detected a reduction of 10–11% in rice production over the course of the past 50 years, provided that the crop’s sowing dates remained constant (64). In contrast, NUCS such as millets, buckwheat, bambara groundnut, and cowpea can adapt to extreme weather (heat and drought stress) with fewer nutrient requirements (65), while cultivation of cereal crops as compared to underutilized crops requires a large agricultural input and contributes significantly to GHG emissions, which furthers global warming (66). Conversely, global warming has a number of negative repercussions on the viability of economies, ecosystems, and agriculture. Underutilized crops usually require less water and thus have high water-use efficiencies compared to staple crops (67). Additionally, they can be cultivated on severely degraded marginal land that is no longer appropriate for high-input commercial crops (67). In India, local finger millet genotypes such as Kurkuti, Lala, Ladu, Jhana, and Taya were shown to outperform other genotypes in terms of their photosynthetic capacity, water-use efficiency, and carboxylation efficiency (68). Pearl millet (*Pennisetum glaucum*) and foxtail millet (*Setaria italica*), mainly cultivated in African and Asian countries, are known for their high salinity and drought tolerance capacities (69), yet they have not been widely adopted due to their low yields and lack of local plant species. Pseudocereals such as buckwheat (*Fagopyrum esculentum* and *Fagopyrum tataricum,* quinoa (*Chenopodium quinoa*), and amaranth (*Amaranthus hypochondriacus*) have been reported to thrive in nutrient-poor soils and to be resilient to various biotic and abiotic stresses (70). Owing to their drought tolerance and ability to thrive in impoverished soil, many countries have started producing cassava (*Manihot esculentum*) and sweet potato (*Ipomoea batata*) as major food sources (71). Hyacinth bean (*Lablab purpureus* L.), Egyptian kidney bean, Indian butter bean, and lablab bean are extremely resilient to drought-prone areas, making them an efficient alternative for protein security (45). Underutilized crops showing different degrees of biotic and abiotic stress tolerance (72, 73) are listed in Supplementary Table 3.

### Anti-nutritional factors in NUCS and abatement strategies

Anti-nutritional factors (ANFs) refer to the phytochemicals that can bind with different nutrients to impede their digestion, absorption, or utilization. If taken in large quantities, these substances can be harmful to human health. Apart from several pharmacognostic properties, some phenolic compounds have been reported to possess anti-nutritional effects on protein metabolism due to their ability to bind to digestion enzymes and protein substrates. ANFs can be broadly categorized into four groups: (1) compounds that affect protein utilization and digestion, such as tannins, lectins, and protease inhibitors; (2) compounds that affect mineral utilization, such as gossypol, phytates, and glucosinolates; (3) antivitamins; and (4) other compounds, such as mycotoxins, cyanogens, alkaloids, mimosine, and saponin. The major ANFs found in some of the selected NUCS are listed in Table 3.

**Table 3:** Anti-Nutritional factors found in NUCS.

Scientific names	Common names	Anti-Nutritional factors	References
*Amaranthus dubias*	Amaranth	Betacyanins, chlorogenic acid, and caffeoyl iso citric acid	(74)
*Fagopyrum esculentum*	Buckwheat	Trypsin inhibitors, phytic acid, and tannins	(75)
*Chenopodium quinoa*	Quinoa	Saponins, phytic acid, oxalates, tannins, and trypsin inhibitors	(76)
*Eleucine coracana*	Finger millets	Tannic acid and phytic acid	(77)
*Pennisetum glaucum*	Pearl millet	Tannic acid and phytic acid	(78)
*Phaseolus* spp.	Pulses	Saponins, glycosides, tannins, alkaloids, phytic acid conjugates, and lectins	(79)
*Vicia faba*	Broad beans	Vicine and convicine	(80)
*Pisum sativum*	Peas	Lectin, tannins, and oligosaccharides	(81)
*Glycine max*	Soybean	Glycinin, lectin, phytic acid, and oligosaccharides	
*Manihot esculenta*	Cassava	Cyanoglucosides	(82)
*Solanum tuberosum*	Potato	Polanines and cyanogens	
*Dioscorea*	Yam	Dioscorine	
*Phaseolus vulgaris*	French beans	Pisatin and phaseottin	
*Colocasia esculenta*	Taro	Oxalate and oxalic acid	
*Fabacea*	Legumes	Antitrypsin factors, trypsin inhibitor, tannins, saponins, amylase inhibitors, protease inhibitors, phytic acids, and lectins	
*Perilla frutescens*	Perilla	Tannic acid and phytic acid	(83)
*Solanum dulcamara*	Nightshade	Oxalate, phytate, nitrate, and alkaloids	(84)
*Annona muricata*	Annona	Phytate and oxalate	(85)
*Artocarpus heterophyllus*	Raw Jackfruit	Tannins, phytate, oxalate, and trypsin inhibitor	(86)
*Phoenix dactylifera*	Dates	Oxalate and tannin	(87)

Although several NUCS-based food products have already been developed, an appropriate processing technology is still mandated to eliminate ANFs such as lectins, α-amylase inhibitor (αAI), and arcelins (Arc) (88). Heat treatment, however, improves their hydrolysis. Recently, an enzyme called VC1 has been characterized that converts GTP to vicine and convicine. Silencing of the VC1 gene or gene editing may be used to develop an anti-nutrient-free faba bean variety (89). The main strategy to remove the anti-nutrient factors could be either by targeting upstream genes or inhibiting the biosynthesis of particular anti-nutrients via different biotechnological routes. The ultrafiltration technique can also be used to remove and separate ANFs into different fractions; however, it does not inactivate ANFs. Considering the feasibility of the technological tools and economic conditions, the soaking of grains followed by heating is an effective strategy to boost protein digestibility and good sensory qualities. While conventional breeding techniques take several years to express desired traits, genome engineering techniques such as CRISPR/Cas9 can be employed to the targeted deletion of anti-nutritional factors of biosynthesis genes and to develop anti-nutrient-free cultivars in lesser time (90).

### Constraints of NUCS: field to food basket

The domestication and cultivation of most neglected and underutilized crops are restricted to their native locations, and so cultivation strategies for large-scale production face many challenges linked to cultivation technology, infrastructure, and market linkage. The poor communication between the scientific community and governments and local farmers is another challenge for the proper utilization and implementation of knowledge. Some of the important constrains of NUCS are listed below:

1. Lack of knowledge and information on the nutritional value, consumption, and utilization of many underutilized plant products that are unpopular when compared to major staple crops.
2. Not enough people are aware of the financial advantages and market opportunities.
3. By using food processing at the village level, standard technology adds value.
4. No appropriate, higher-quality planting material is used, and no breeding or biotechnology efforts are made to shorten gestation times and increase fruit production.
5. The researchers, agriculturalists, and extension personnel showing less interest.
6. Poor producer interest and yield.
7. Losses during post-harvest and transportation.
8. For underutilized fruits, there is no infrastructure or marketing network.
9. The nation lacks proper credit, investment, and policy.
10. Insufficient scientific resources are available for evaluating, testing, and post-harvest management of various underutilized fruits.

### Way forward to overcome challenges of NUCS

Several distinct approaches can be suggested as prospective solutions for the partial or complete overcoming of the major constraints of NUCS. Some of them are highlighted below:

1. Well-organized exploration programs and ecogeographic surveys must be carried out in order to create a database on the origin, distribution, habitat, agroclimatic requirements, advantages, and scientific application of potential underutilized crops.
2. In order to maximize the potential of underutilized crops, more focus must be given to developing suitable plant types with traits such as early emergence, photo insensitivity, high harvest index, lodging and shattering resistance, and determinate, bushy growth habit. Short-duration cultivars must be improved to work effectively in current farming systems and to thrive in unconventional seasons and locations.
3. Effective agronomic management is needed to incorporate underutilized crops into current agricultural systems. To ascertain the bundle of agricultural practices relating to sowing time and manner, seed quality, plant density and arrangement, irrigation, fertilization, and harvesting in various crops, well-prepared experiments are required.
4. More emphasis needs to be placed on in-depth research on nutritional quality, nutraceutical qualities, and anti-nutritional elements. Processing, value addition, product creation, and effective marketing strategies also require more attention.
5. To keep scientists, extension agents, and farmers informed of the most recent technological advancements pertaining to certain crops, training programs need to be held on a regular basis.
6. To create an efficient value chain to encourage the use of these potentially underutilized crops, close ties between growers, traders, processors, consumers, and other formal and informal sectors must be developed.
7. Priority should be given to adopting policies that will mainstream the use of neglected and underutilized crop species in food systems.
8. In order to give the essential impetus to research and development activities on underutilized crops, non-governmental organizations (NGOs) should be involved at the relevant levels.

### Global/national efforts to improve NUCS productivity to fight global hidden hunger

**Figure 3:**
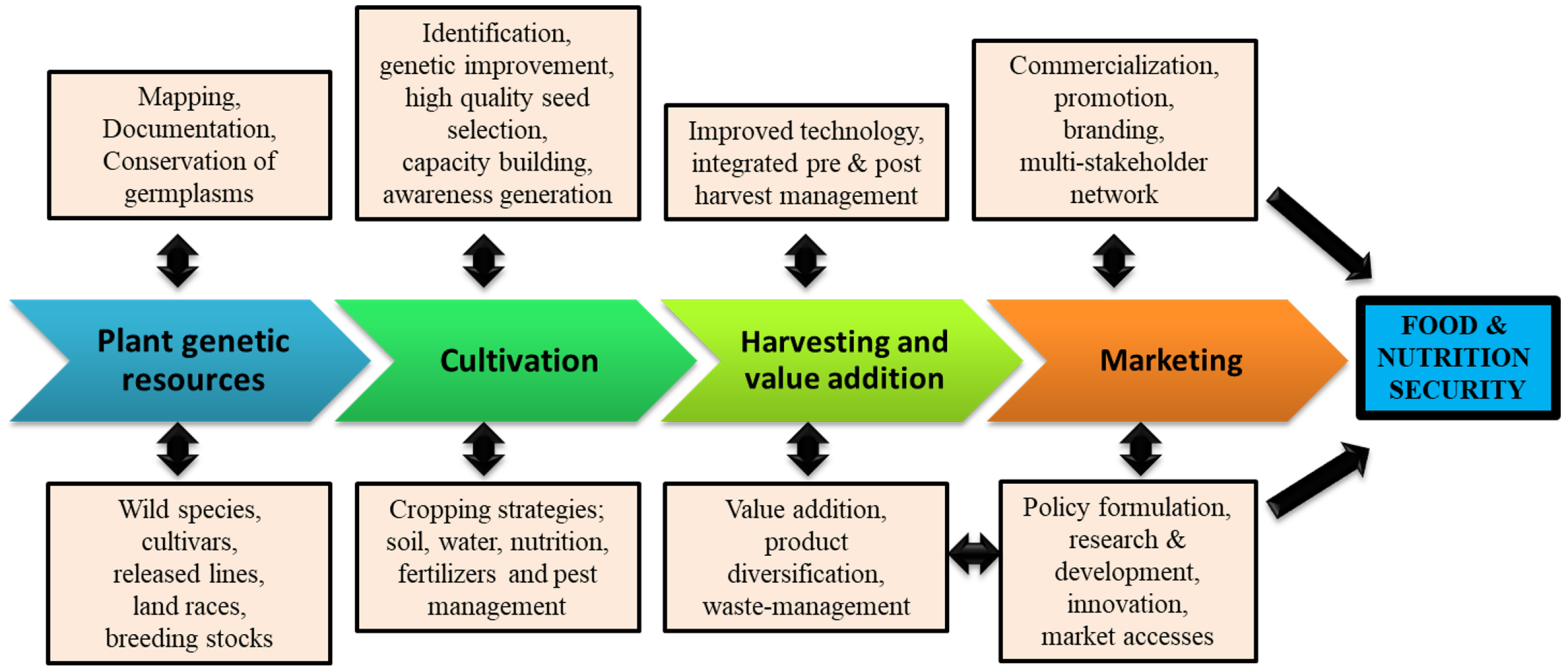
Value chain framework of NUCS.

While the majority of organizations and research and development NGOs and INGOs have primarily focused on the mandate cereal crops, very few initiatives have focused on NUCS. Recently, emphasis has been laid on the underutilized crops that have enormous potential, a range of nutrient statuses, and that require very little effort to incorporate into sustainable agriculture systems. NUCS have been considered under several projects by the SDGs, International Treaty on Plant Genetic Resources for Food and Agriculture (ITPGRFA), biodiversity conservation plans, and the UN for sustainability issues, etc. These underutilized crops are now improving the sustainability of marginal local communities, as seen in India through the IFAD’s kodo and millet program, in the Andes through its grain-centric program, in Mali by the bambara groundnut and fonio program, and in Guatemala through the tepary bean and Mayan spinach project (91). LI-BIRD, SAHAS, Helvetas, FAO, NARC, and Bioversity International have been working on a few of the neglected and underutilized crops such as amaranth, finger millet, buckwheat, beans, proso millet, foxtail millet, barley, yam, and turnip (92). The last decade was marked by a remarkable increase in support for NUCS from overseas development agencies (ODA), the IDRC, the Asian Development Bank, the European Commission, and several other donors joined by Germany and the United Kingdom in financing *ad hoc* projects and networks dealing with NUCS. Some of the important networks include BAMNET (International Bambara Groundnut Network), MEDUSA (Network on the Identification, Conservation and Use of Wild Plants in the Mediterranean Region), PROSEA (Plant Resources of South East Asia), UTFANET (Underutilized Tropical Fruit in Asia Network), and SEANUC (Southern and East Africa Network on Underutilized Crops) (93). The United States Department of Agriculture (94) has also implemented a project on climate-resilient orphan crops for increased diversity in agriculture. The project aims at reinforcing agro-biodiversity in distinct socio-economic and geographic locations with three major objectives: the promotion of six important underutilized arable crops, namely, oats, hull-less barley, triticale, buckwheat, faba bean, and lupin; the development of value chains for particular underutilized crops; and the analysis of the project result’s socioeconomic effects. This concept is a cutting-edge, problem-driven strategy built on the promotion of underutilized crops in eco-sustainable cropping systems and locally sourced value chains (Figure 3).

In the current scenario, the world is facing three major intertwined challenges, *viz.*, climate change, food security, and sustainability. As discussed above, owing to their nutrient-dense and climate-resilient properties, expanding the use of these crops would boost agricultural biodiversity (in respect to genes, species, and ecosystems) to protect crops from climate change, pests, and diseases and would provide a wide range of high-quality food sources for ensuring food and nutritional security. For a household to be considered food secure, its members must always have access to adequate food to lead active and healthy lives. At a minimum, food security entails the immediate availability of nutrient-dense and safe foods and the assurance of being able to obtain appropriate foods in socially acceptable ways. While the conceptual framework of nutrition security strives to better comprehend whether dietary-related diseases and inequities coexist with food scarcity—specifically in the cases of people who belong to members of racial or ethnic minorities, those with lower incomes, and those who live in rural or distant areas—nutrition security involves having consistent access to, availability of, and affordability of foods and beverages that promote health and prevent disease. A total of 50% of the world’s plant-based calorie intake supplied from only three crops (i.e., wheat, rice, and maize), the production of which covers around 40% of arable land. However, these crops alone cannot provide sufficient nutrients, and therefore, a diverse diet is required that can be accessible by the poorest. This is why it is time to look into some of the potential food crops (NUCS) that are thought to exist globally. The food system as we currently know must undergo a drastic revolution. In particular, underutilized plant-based foods can be excellent sources of macro and micronutrients; legumes abundant in protein, dietary fiber, and trace elements are only a few notable examples. Improvising in the agricultural extension of these crops would enhance the economic growth of countries and present a step forward to eradicate poverty. Under the SDGs, there are 17 aspirational “Global Goals” and 169 targets. Three of these objectives are specifically related to agriculture. These are “Climate Action,” “Zero Hunger,” and “No Poverty.” Therefore, each country must focus on the three key goals of food, nutrition, and healthcare. The debate surrounding underutilized crops frequently centers on how well suited these crops are to places of sparse output, with a special focus on such crops in developing countries. In this regard, it is claimed that if promoted in certain regions, they may have a favorable effect on food production. As a result, household earnings, food security, and nutrition would all increase. Considering this, it becomes apparent that underutilized crops may be able to serve certain SDGs. Therefore, acceptance and enhancing the agro-diversification of these food crops (NUCS) could be one of the answers to achieve zero hunger and fulfill the SDGs more quickly.

## Concluding remark and policy implications

Regardless of agricultural and technological advances, malnutrition, hunger, and food insecurity remain serious issues in the present world. Limited dietary diversity and persistent malnutrition are mostly caused by an overreliance on basic crops. Although NUCS have the potential to fight hidden hunger and malnutrition, underutilized crops are still ignored, specifically NUCS. Future smart food crops have four distinct advantages over staple crops that could help the world to achieve zero hunger: nutrition, climatic resilience, local relevance, and economic feasibility. Different studies on the nutraceutical and pharmacognostic properties of NUCS have revealed presence of good proteins, dietary fibers, minerals, polyphenols, vitamins, and other bioactive compounds. The bioactive compounds in NUCS have the potential to combat chronic ailments such as cancer, neurological disorders, cardiovascular diseases, hypertension, or diabetes (15, 16, 95, 96). Despite their several nutrition-rich qualities, improved cultivation strategies for the mass production of these crops are still underdeveloped. Meanwhile, the scientific knowledge acquired from different research fields mostly remains within the scientific community. Therefore, an effective network system between farmers, researchers, entrepreneurs, and governments is very crucial. Additionally, the proper execution of NUCS-based INGOs/NGOs and government policies still requires focus. Some of the important global/national events that have fostered NUCS are listed in Supplementary Table 4.

It is evident that most agricultural and food policies are based on a limited number of staple crops. Therefore, it is recommended that every country should have a particular policy to ensure the promotion and usage of underutilized crops. Although the National Department of Agriculture, Forestry and Fisheries (DAFF) Strategic Plan for 2016–2020, the National Policy on Food and Nutrition Security (97), and the National Plan on Integrated Growth and Development Planning (IGDP-2010) are all in sync with the strategy for promoting NUCS, the challenge of the proper execution of these policies with reference to NUCS has remained ambiguous. Therefore, policies that facilitate the mainstreaming of the use of underutilized crops in food systems require special attention as follows:

1. Given their excellent nutritional value, it is important to encourage children to eat NUCS-based food products, especially as part of their midday meals.
2. Farmers should be granted the proper subsidies to encourage them to grow underutilized crops. Reducing the price of high-quality seed is necessary, and the initial procurement of their output must be guaranteed.
3. To promote the spread of an already well-known but underutilized crop, local political and administrative support must be built. In order to cultivate these crops, governing bodies must be persuaded through appropriate discussion. By giving those rewards and subsidies, farmers should be encouraged to plant the underutilized crops.
4. In order to give the essential impetus to research and development activities on NUCS, non-governmental organizations (NGOs) should be involved at the relevant levels.
5. To strengthen research and development programs on NUCS, adequate resources, both financial and manpower, must be allocated.
6. The free exchange policies of climate-resilient germplasms within or between nations should be emphasized.

To achieve these goals, policy packages should be designed in a coherent manner, each with a clear objective and a set of targets that emphasize accountability for execution. In India, a notable approach taken in Chhattisgarh state, under the Pradhan Mantri Poshan Shakti Yojana, is the adoption of millet-based foods as a midday meal for school children. The implementation of this policy is needed in other states as well. The Ministry of Agriculture and Farmers Welfare, India, under different central schemes such as ATMA, AGMARKNET, Pradhanmantri Krishi Sinchayee Yojana, mKisan, Pradhan Mantri Fasal Bima Yojana, and Kisan Call Center, has targeted the improvement of crops and agro-diversification of nutraceutical crops. The inclusion of NUCS under these schemes for mass cultivation would improve the nutrition security of the country. MOVCD-NER (Mission Organic Value Chain Development for North East Region) is another central sector scheme under the National Mission for Sustainable Agriculture (NMSA), launched by the Ministry of Agriculture and Farmers Welfare for implementation in the states of Arunachal Pradesh, Assam, Manipur, Meghalaya, Mizoram, Nagaland, Sikkim, and Tripura. This program aims to support the growth of the entire value chain, from inputs and distribution of seeds to the certification and establishment of facilities for collection, aggregation, processing, marketing, and branding. It also aims to develop certified organic production in a value chain to connect farmers and consumers. Currently, the center is working on an improved roadmap to promote the cultivation of crops, particularly okra, gourd, ginger, pineapple, and red rice. Similarly in Africa, the National Food and Nutrition Security Policy 2014 (97), which aims to ensure food availability, accessibility, and affordability, could be achieved by integrating NUCS, which are locally available, nutritious, and the cheapest source of macro and micronutrients. Incorporating NUCS into the policies of the 2014–2019 National Climate Change and Health Adaptation Plan has proven helpful in maintaining socioeconomic and environmental resilience due to their sustainability and health benefits.

In conclusion, since most of the available research projects on NUCS are typically dispersed and difficult to assemble in one research article, this article attempts to address the background information on the proper identification of NUCS and their nutrient content, nutraceutical properties, and major constraints. Furthermore, we enumerated potential strategies to overcome these challenges and global/national policies for improvising NUCS to eradicate global hidden hunger.

## Author contributions

AA and BB conceived the concept of the manuscript. AA wrote the manuscript. BB supervised the manuscript. All authors contributed to the article and approved the submitted version.

## Conflict of interest

The authors declare that the research was conducted in the absence of any commercial or financial relationships that could be construed as a potential conflict of interest.

## Publisher’s note

All claims expressed in this article are solely those of the authors and do not necessarily represent those of their affiliated organizations, or those of the publisher, the editors and the reviewers. Any product that may be evaluated in this article, or claim that may be made by its manufacturer, is not guaranteed or endorsed by the publisher.
