# Supplementary material for: Nutrition security, constraints, and agro-diversification strategies of neglected and underutilized crops to fight global hidden hunger
Source: Front Nutr. 2023 Jun 22;10:1144439. doi: 10.3389/fnut.2023.1144439 (PMC10324569; doi:10.3389/fnut.2023.1144439)
Supplement: Supplementary file 1 [file Data_Sheet_1.pdf]

Source Information and Methodology

In the present review article we provided a comprehensive comparative nutrient profile of staple crops vs. potent underutilized crops with reference to the climate resilience. Major bioactive components, bioavailability, Anti-nutritional factors, along with different constrains and way forward of NUCS were also briefly discussed.

The articles for the literature study were gathered using a multi-step procedure. We used different academic research databases such as Scopus, JSTOR, Web of Science, EBSCO, Google Scholar, ScienceDirect, PubMed and Academic Search. Each database was searched using following search queries:

1. “Neglected and underutilized crops”, “NUCS” and “NUS”
2. “Pseudocereal” and “Nutrition value”
3. “Antinutrition factors in Neglected and underutilized crops”
4. “Sustainable development goal”
5. “USDA and NUCS”
6. “Food security”
7. “Identification of NUCS”
8. “Bioactive components of NUCS”
9. “Climate resilient crops” and “NUCS”
10. “Constraints of NUCS”
11. “Way forward to overcome constraints of NUCS”
12. “Govt./NGO policies in NUCS”
13. “Health benefits of NUCS”
14. We also used scientific and common name of each species mentioned in this article as query to find relevant information available in the databases.

These queries produced a hit of 2345 article. Only research and review publications were the primary emphasis of the first stage. The initial reduction left 437 papers after all hits that did not pertain to the chosen research area that focus on how NUCS can be an answer to nutrition security, different constraints of NUCS and global/national efforts to improvise NUCS to fight global hidden hunger**.** The next stage was to eliminate all duplicate publications, leaving 214 publications for further examination. The 214 articles' abstracts were examined as the last step in the selection process to get a general idea of what they were about. After cross-checking we further concentrated on 195 research and review articles that we had to read in full to see if they answered any of our study topics. Following this reading, 117 articles that gave only background information and similar or duplicating results were discarded leaving 96 pertinent papers responding to the research questions in our study. Primary emphasis has been given to most recent publications, especially of 2019 to 2023.
