# Supplementary material for: Nutrition security, constraints, and agro-diversification strategies of neglected and underutilized crops to fight global hidden hunger
Source: Front Nutr. 2023 Jun 22;10:1144439. doi: 10.3389/fnut.2023.1144439 (PMC10324569; doi:10.3389/fnut.2023.1144439)
Supplement: Supplementary file 2 [file Table_1.pdf]

Supplementary Table 1: List of potent Neglected and Underutilised Crop Species (6, 10, 12)

NUCS categories	Scientific Names of the NUCS
Cereals and pseudocereals	*Amaranthus caudatus, Chenopodium pallidicaule, C. quinoa, Digitaria exilis, Eleusine coracana, Echinochloa frumentacea/ utilis, Fagopyrum esculentum, F. tataricum, Panicum miliaceum, Panicum miliare, Setaria italica, Paspalum scrobiculatum, Triticale*
Fruits and nuts	*Annona muricata, A. cherimola/ muricata, Aeglos marmelos, Artocarpus heterophyllus, Anacardium occidentale, Adansonia digatata, Averrhoa carambola, Bactris gasipaes, Blighia sapida, Borassus aethiopicum, Balanites aegyptiaca, Carica papaya, C. pubescens and hybrids, Canarium spp., Citrus grandis, Carya cathayensis, Cyphomandra betacea, Couepia longipendula, Diospyros mespiliforme, Dimocarpus longan, Durio zibethinus, Dovyalis spp., Embilica officinalis, Eugenia stipitata, Euterpe oleracea, Feijoa sellowiana, Garcinia mangostana, Hovenia dulcis, Juglans, Litchi chinensis, Manilkara zapota, Nephelium lappaceum, Opuntia ficusindica, Passiflora edulis/ quadrangularis, P. mollissima, P. ligularis, Psidium guajava, P. angulatum, Paullinia cupana, P. Salacca zalacca, Sclerocarya birrea, Strychnos spp. Solanum muricatum, S. quitoense, Theobroma grandiflorum, Terminalia koernbachii, Tamarindus indica, Uapaca spp. , Vitis spp., Vitellaria paradoxa, Zizyphus mauritiana,*
Vegetables and pulses	*Amaranthus spp., Anaranthus spp., Bidens pilosa, Brassica carinata, Basella alba, Bactris hearts, Basella rubra, Bauhinia purpurea, Butea monosperma, Curcuma spp., Celosia spp., Cnidoscolus chayamansa, Cucurbita spp. espec.* Squashes, *Caesalpinia crista, Canavalia ensiformis, Crotalaria spp., Corchorus spp., Cucurbita spp., Citrullus local spp., Chamaedorea tepoiilote hearts, Colocasia/ Xanthosoma leaves, Crambe cordifolia, Chenopodium album, Divers local bamboo spp., Euterpe hearts, Emilia spp., Gymnandropsis synandra, Gliricidia sepium, Glycine* Vegetable, *Hibiscus sabdariffa, Ipmoea aquatic, Lablab purpureus, Lagenaria spp., Lupinus mutabilis, Lathyrus spp., Lagenaria spp., Macrotyloma uniflorum, Millettia pinnata, Mimosa pudica, Momordica spp., Mucuna spp., Moringa oleifera, Mucuna spp., Opuntia spp., Psophocarpus tetragonolobus, Phanera vahlii, Parkia roxburghii, Physalis philadelphicus, Portulaca oleracea, Pachyrhizus spp., Phytolocca acinosa, Parkia biglobosa, Rorripa indica, Solanum americanum/ nigrum, S. aethiopicum, S. macrocarpus, Talinum triangulare, Vigna umbellate, V. subterranean, V. aconitifolia, V. angularis, V. umbellate, Voandzeia subterranean, Vernonia spp.,*
Roots and tubers	*Alocasia spp., Amorphophallus paeoniifolius, Arracacia xanthorrhiza, Calathea allouia, Canna edulis, Colocasia esculenta, Dioscorea spp., Harpagophytum procumbens, Ipomoea batatas, Manihot esculenta, Oxalis tuberosa, Pachyrhizus erosus/tuberosus Plectranthus esculentus, Sphenostylis stenocarpa, Solenostemon rotundifolius, Tylosema fassoglense, T. esculentum, Tropaeolum tuberosum, Ullucus tuberosus, Vigna vexillata, Xanthosoma sagittifolium,*
