# Supplementary material for: Nutrition security, constraints, and agro-diversification strategies of neglected and underutilized crops to fight global hidden hunger
Source: Front Nutr. 2023 Jun 22;10:1144439. doi: 10.3389/fnut.2023.1144439 (PMC10324569; doi:10.3389/fnut.2023.1144439)
Supplement: Supplementary file 3 [file Table_2.pdf]

Supplementary table 2: Amino acid composition (g/100g) in selected cereal crops and Neglected and Underutilised Crop Species (10, 19, 95, 96)

Common name	Scientific name	Amino acids (g/100g dry weight)														
		Alanine	Arginine	Aspartate	Glutamate	Glycine	Histidine	Isoleucine	Leucine	Lysine	Methionine	Phenylalanine	Serine	Threonine	Tyrosine	Proline
Rice	*Oryza sativa*	1.20	1.20	1.40	2.20	0.90	0.50	0.60	1.40	1.00	0.10	0.90	0.70	0.60	0.50	0.60
Wheat	*Triticum aestivum*	0.31	0.46	0.42	3.53	0.37	0.24	0.38	0.73	0.22	0.22	0.53	0.49	0.28	0.34	1.28
Maize	*Zea mays*	0.89	0.50	0.74	0.51	0.40	0.31	0.40	1.50	0.31	0.24	0.69	0.57	0.34	0.51	0.91
Finger Millet	*Eleucine coracana*	0.58	0.38	0.57	0.52	0.33	0.23	0.39	0.93	0.24	0.28	0.49	0.48	0.39	0.56	0.66
Pearl Millets	*Pennisetum glaucum*	0.75	0.43	0.76	0.55	0.35	0.25	0.41	0.98	0.28	0.24	0.48	0.44	0.38	0.53	0.67
Foxtail Millets	*Setaria italic*	0.59	0.93	0.30	2.20	0.29	0.21	0.55	1.36	0.16	0.31	0.63	0.46	0.37	0.24	0.55
Buckwheat	*Fagopyrum esculentum*	0.54	1.40	1.30	2.50	0.83	0.26	0.40	0.67	0.60	0.23	0.48	0.70	0.40	0.70	0.54
Amaranth	*Amaranthus caudatus*	0.53	1.47	1.22	2.51	1.37	0.38	0.55	0.86	0.83	0.34	0.61	0.88	0.43	0.54	0.69
Quinoa	*Chenopodium quinoa*	0.53	1.27	1.07	1.89	0.88	0.36	0.48	0.84	0.70	0.31	0.50	0.57	0.37	0.41	0.56
Hyacinth beans	*Lablab purpureus*	0.33	0.05	0.57	1.30	0.30	2.20	15.00	2.50	5.20	0.13	3.50	0.15	0.12	1.70	0.60
Pea	*Pisum sativum*	0.40	0.79	0.96	1.86	0.24	0.26	0.54	0.80	0.24	0.07	0.53	0.37	0.27	0.47	0.24
Faba Beans	*Vicia faba*	0.41	0.84	1.05	1.90	0.24	0.32	0.55	0.84	0.22	0.07	0.57	0.39	0.29	0.47	0.21
Soy Beans	*Glycine max*	0.39	0.62	0.94	1.78	0.40	0.26	0.53	0.51	0.27	0.11	0.51	0.37	0.40	0.42	0.23
Jack Fruit	*Artocarpus heterophyllus*	0.24	1.25	0.25	2.67	2.67	0.10	0.16	0.40	0.24	0.20	0.21	0.31	0.39	0.50	2.59
Kidney Beans	*Phaseolus vulgaris*	0.39	0.45	1.13	2.12	0.33	0.31	0.35	0.90	0.74	0.16	0.31	0.58	0.30	0.32	0.48
Rice Bean	*Vigna umbelata*	1.03	1.25	2.48	3.48	0.87	0.64	1.03	1.22	1.64	2.10	12.60	8.80	7.30	5.30	9.30
Perilla	*Perilla fretescens*	0.10	0.13	0.25	0.35	0.09	0.06	3.69	6.86	4.38	2.34	0.52	0.65	2.20	0.17	0.32
Adzuki Beans	*Vigna angularis*	0.41	0.78	1.13	1.77	0.37	0.36	0.50	0.87	0.85	0.18	0.63	0.45	0.37	0.33	0.55
Lima Beans	*Phaseulus lunatus*	0.40	0.48	1.01	1.10	0.33	0.24	0.41	0.67	0.52	0.10	0.45	0.52	0.34	0.28	0.35
Cassava	*Manihot esculenta*	3.31	3.23	3.31	3.92	2.40	1.08	3.13	4.17	2.42	0.54	2.82	1.59	2.27	1.99	1.62
Yam	*Dioscorea escuenta*	3.31	3.15	3.31	3.75	2.41	0.95	1.70	3.58	2.36	0.37	2.82	1.41	2.16	1.90	1.39
Nightshade	*Solanum dulcamara*	0.16	0.13	0.53	0.12	0.35	0.17	0.28	0.18	0.09	0.17	3.09	0.14	0.26	0.03	0.23
