# Supplementary material for: Nutrition security, constraints, and agro-diversification strategies of neglected and underutilized crops to fight global hidden hunger
Source: Front Nutr. 2023 Jun 22;10:1144439. doi: 10.3389/fnut.2023.1144439 (PMC10324569; doi:10.3389/fnut.2023.1144439)
Supplement: Supplementary file 4 [file Table_3.pdf]

Supplementary table 3: List of Neglected and Underutilized Crop Species (NUCS) showing tolerance to biotic or abiotic stress (71, 72)

Family	Scientific Name	Common Name	Nutritive property
Amaranthaceae	*Amaranthus retroflexus*	Amaranthus	Grains rich in protein, (13–19%), rich squalene and antioxidants
	Chenopodium pallidicaule	Canihua	Excellent source of protein, carbohydrate fat, and ash content
	Salicornia bigelovii	Salicornia	Rich in bioactive compounds, vitamin A, minerals and fatty acids. Seed oil rich in linoleic acid
	Chenopodium quinoa	Quinoa	Gluten-free, with high protein concent, rich in unsaturated fatty acids, vitamins, and minerals
Brassicaceae	*Camelina sativa*	Camelina	Excellent source of essential unsaturated fatty acids, specially, OMEGA-3 fatty acids
	Brassica juncea	Indian mustard	Seeds rich in glucosinolates, sterols, and phenols. Leafs rich in glucose, fructose, and minerals
Convolvulaceae	*Ipomoea batatas*	Sweet potato	Protein content ranging from 4–27%, rich in β-carotene and anthocyanin
Fabaceae	*Vigna unguiculata*	Cowpea	High in protein (<20%) and minerals like calcium, potassium, sodium, etc
	Vigna subterranea	Bambara ground nut	Excellent source of protein, unsaturated fatty acids and essential minerals
	Cyamopsis tetragonolobus	Guar	High polyphenol, protein, ash, and contents
	Lablab purpureus	Lablab	Rich in carbohydrates, proteins, vitamins minerals and
	Cajanus cajan	Pigeon pea	Good source of protein, starch, crude fibre, fat, minerals, calcium and manganese,
	Sesbania sp.	Sesbania	Excellent source of protein content (can exced 40%), calcium and vitamin C
	Lupinus albus	White lupin	Good protein source, fatty acid, fibre content, antioxidants, and non-starch

			carbohydrates
Lamiaceae	*Salvia hispanica*	Chia	Seeds with high protein content (>15%), rich in fatty acids, and minerals.
Poaceae	*Triticum spelta*	Spelt	Higher protein content, less lysine and more non-essential amino acids
	Eragrostis tef	Tef	Gluten-free, polyphenols, rich in protein, dietary fiber, and minerals
	×*Triticosecale*	Triticale	Rich in protein content and more nutritious than wheat
	Tritordeum martinii	Tritordeum	Rich in, dietary fiber content, free amino acids total phenol content and antioxidant activity
Polygonaceae	*Fagopyrum esculentum*	Buckwheat	Rich source of flavonols like rutin, high antioxidant, higher protein content and gluten free
Portulacaceae	*Portulaca oleracea*	Purslane	Rich in omega-3 acids, amino acids, and vitamins
Ranunculaceae	*Nigella sativa*	Nigella	Good source of fatty acids, glycolipids, phytosterols, and phospholipids
