# Supplementary material for: Nutrition security, constraints, and agro-diversification strategies of neglected and underutilized crops to fight global hidden hunger
Source: Front Nutr. 2023 Jun 22;10:1144439. doi: 10.3389/fnut.2023.1144439 (PMC10324569; doi:10.3389/fnut.2023.1144439)
Supplement: Supplementary file 5 [file Table_4.pdf]

Supplementary table 4: Significant global events/projects fostering NUCS

Crops	Year	Events/ projects
Underutilized Crops	1982	All India Coordinated Research Project (AICRP) on Under-utilized plants (UUP) in India
	1987	1987 - Establishment of the International Centre for Underutilized Crops (ICUC)
	1997	The FAO Global Plan of Action for Plant Genetic Resources for Food and Agriculture emphasized the importance of underutilized crops
	1999	At an international workshop held in Chennai, India, the Consultative Group of International Agricultural Research (CGIAR) recognized the contribution that neglected and underutilized species make to food security, rural incomes and combating poverty
	2002	Establishment of the Global Facilitation Unit of Underutilized Species (GFU) of the Global Forum on Agricultural Research (GFAR), and was housed within Bioversity International, Rome, Italy.
	2005-2006	Mission on Integrated Development of Horticulture (MIDH, then 'National Horticulture Mission', NHM) gave special impetus to establish orchards of underutilized fruit species in India
	2008	Establishment of Crops for the Future (CFF) which is a merging of ICUC and GFU, based in Malaysia
	2011	Establishment of Crops for the Future Research Centre (CFFRC) in Malaysia
	2012	The international *Crops for the 21st Century* seminar held in Córdoba, Spain aimed to discuss major topics related to underlining the role of neglected and underutilised species to address food and agriculture challenges in the future
Andean grains, minor millet & medicinal and aromatic plants	2001 - 2010	Empowering the rural poor by strengthening their identity, income opportunities and nutritional security through the improved use and marketing of neglected and underutilized species (IFAD NUS I & II) in Bolivia, Ecuador, Peru, India, Nepal, Egypt & Yemen
Winter vegetables, minor millet & Andean grain	2011 - 2015	Reinforcing the resilience of poor rural communities in the face of food insecurity, poverty and climate change through on-farm conservation of local agrobiodiversity (IFAD NUS III & IV) in Nepal, India & Bolivia
underutilized crops	2013	3rd International Conference on Neglected and Underutilized Species (NUS): For a Food-Secure Africa in Accra, Ghana
Quinoa	2013	Official launch of the International Year of Quinoa (IYQ-2013), intended to increase awareness, understanding and knowledge about quinoa and its importance on food security
Bambara groundnut & amaranth	2014-2016	Strengthening capacities and informing policies for developing value chains of neglected and underutilized crops in Africa (ACP-EU) in Benin, Kenya & Zimbabwe
190 indigenous and wild	2014 - present	Biodiversity for Food and Nutrition in Brazil, Kenya, Sri Lanka & Turkey

edible species		
Buckwheat, cold tolerant rice, common bean, finger millet, foxtail millet, grain amaranth, naked barley & proso millet.	2014 - present	Integrating Traditional Crop Diversity for Mountain Food security in Nepal
Bambara groundnut, fonio, chaya, tepary bean & minor millet	2015 - present	Linking agrobiodiversity value chains, climate adaptation and nutrition: Empowering the poor to manage risk (IFAD-EU NUS) in Mali, Guatemala & India
Millets	2023	Official launch of International Year of Millets (IYoM-2023) in India with a theme to “Harnessing the untapped potential of millets for food security, nutrition, and sustainable agriculture”
